# Role of Nutrition and Diet on Healthy Mental State

**DOI:** 10.3390/nu14040750

**Published:** 2022-02-10

**Authors:** Roser Granero

**Affiliations:** 1 Department of Psychobiology and Methodology, Autonomous University of Barcelona, 08193 Barcelona, Spain; Roser.granero@uab.cat; 2 Ciber Fisiopatología Obesidad y Nutrición (CIBERobn), Instituto Salud Carlos III, 28015 Madrid, Spain; 3 Psychoneurobiology of Eating and Addictive Behaviors Group, Neurosciences Programme, Bellvitge Biomedical Research Institute (IDIBELL), 08908 Barcelona, Spain

## 1. Introduction

A large number of scientists and health professionals recognize that balanced nutrition is fundamental for a good state of physical health. The World Health Organization working group focused on nutrition as a key component of disease prevention, indicating that “*a balanced and varied diet, composed of a wide range of nutritious and tasty foods, adds years to life and life to years*” [1]. In their report, this group also warns that a high percentage of common diseases in industrialized countries (such as obesity, diabetes, hypertension, coronary heart disease and even certain cancers) are directly or indirectly related to inefficient nutrition, especially with the elevated intake of processed foods high in trans-fatty acids and low consumption of essential nutrients (mainly vitamins, minerals and proteins). The 13th General Programme of Work approved by the Health Assembly during May 2018 (GPW13) [2], was developed to guide the work team of WHO during 2019–2023, providing priority actions to promote wellbeing during the lifetime (the key element being the reduction of salt/sodium intake and industrialy produced trans-fat) and encouraging the support of the Member States with a roadmap for countries [3].

However, a greater controversy exists in the scientific community regarding the role of nutrition on the onset and progression of mental diseases and behavioral problems, and it is unclear how diet may contribute to therapeutic efficiency regarding patients with diverse psychopathological states. Unfortunately, strategies based on making diet changes and sticking with them are often overlooked in treatments for mental health conditions.

In the following sections, we review current studies that analyze the role of specific diet components in the interventions addressed for common mental disorders in developed countries among children and adolescents. Since the psychopathologies considered in this review have elevated risk of comorbid health hazards, the evidence-based interventions for psychiatric patients covering proper nutrition could promote large-scale physical and mental wellbeing. Furthermore, the results of these intervention programs could provide the basis for developing targeted disease prevention programs aimed to reduce modifiable risk factors.

## 2. Diet Intervention on ADHD

Attention-deficit/hyperactivity disorder (ADHD) is a chronic neurodevelopmental disorder whose etiology is the result of complex interactions between multiple factors, including genetic, biological and environmental influences. The disorder is usually diagnosed when a child is of school age, with a worldwide prevalence estimate of 6% during childhood [4], and persistent into adulthood with a mean rate of 43% (estimates between adults is around 3% within population-based samples) [5]. Several harmful consequences are associated with ADHD, including deficient academic/work performance, social isolation, aggressive behavior (including delinquency and illegal acts) and even premature death from unnatural causes (such as accidents) [6].

The standard intervention for ADHD combines pharmacological treatment (largely psychostimulants) with psychological therapy. The beneficial short-term efficacy of such treatments in reducing acute core symptoms is largely verified, but the long-term effects are not clearly evidenced: most patients may still show ADHD symptoms and may not attain normalized behavior even with combined medication and behavioral therapy, which results in frequent medication nonadherence (around 50% within months 12 to 36 of the follow-up). Current studies on alternative interventions for ADHD aim at the prevention of ADHD progression and targeting the underlying triggers (such as stress, poor sleep, overstimulation, technology or dietary plans). On the basis that making adequate lifestyle changes to minimize these triggers could contribute to better control of ADHD symptoms, studies addressing the efficacy of nutrition on the developmental course of ADHD have observed that deficiencies in certain types of foods can worsen the symptoms of attention deficit, hyperactivity and impulsivity, while adequate dietary plans could optimize brain functions. Most of these treatment studies are focused on exploring the role of vitamins, minerals and polyunsaturated fatty acids [7], with controversial results depending on diverse factors (i.e., sample composition, measurement tools or diet components).

Results obtained by a current systematic review reinforce the effectiveness of ADHD treatments with complementary diet interventions, although the benefits could be different for subgroups of patients with different profiles. Concretely, the study of Granero and colleagues [8] focused on the contribution of iron and zinc supplementation in the progression of ADHD among children and adolescents, observed in randomized trials published during the last two decades. The conclusion was that at baseline (before the treatment), low zinc and iron levels were associated with higher symptom levels (particularly with attention capacity and hyperactivity behavior), suggesting a pathway mediated by the dopaminergic system. Additionally, regarding the contribution to the treatment of ADHD, it was observed that zinc supplementation consistently improved and compensated baseline borderline zinc nutrition, contributing to improvements in most ADHD cases. However, the role of iron supplementation was more inconsistent, and it seemed that its benefits are centered specifically in children with low ferritin/iron stores at baseline, as restoring adequate levels contributes to optimizing the response to psychostimulants used as medical intervention.

The systematic review of Händel et al. was centered around assessing the contribution of using polyunsaturated fatty acids (PUFAs, concretely omega 3 and omega 6) in the treatment plans of ADHD children and adolescents, obtained from randomized clinical trials [9]. Based on a substantial body of evidence, this review concluded that the benefits of PUFAs were not clear on core symptoms of ADHD reported by parents and teachers, neither were they clear on other behavioral measures nor on quality of life measures. However, the authors’ advise that these global results should be considered with caution due to discrepancies regarding their methodologies and assessment tools.

An open trial conducted by Yorgidis and colleagues explored the role of oligoantigenic diet (OD) as a therapeutic tool within ADHD children [10] and observed that the combination of this diet and the subsequent food challenge was efficient to identify individual food sensitivity in connection with ADHD. OD is an individualized type of elimination diet aimed at identifying specific foods that worsen psychopathological symptoms, followed by reintroducing other alternative foods that improve neuropsychological performance. The results observed in the study of Yorgidis are relevant considering that some treatment guidelines on ADHD simply recommend restricted/elimination diets among ADHD children with a history of adverse reactions to specific (groups of) foods without providing alternative dietary plans. The basis of the OD is the identification of high food sensitivity in ADHD children, with the objective of including individualized dietary plans within multimodal therapies. Other current studies have also observed that this is a promising approach for decreasing ADHD symptoms [11,12].

On the other hand, some studies have also assessed the role of caffeine exposure within the ADHD profile and as a therapeutic tool. Since it was observed that both chronic and acute exposure to caffeine impacts the central nervous system modulating neuronal pathways, the possible existence of a link between the modulation of these neurotransmitters and the development/attenuation of neuropsychiatric outcomes is supposed. Animal research has observed that non-toxic doses of caffeine elicit neuro-pharmacological actions by blocking adenosine A receptors in the brain, which leads to the blockade of adenosine kinase and a decrease in the release of adenosine [13]. Since adenosine A2 receptor is a G-protein coupled receptor that regulates several functions in the central nervous system (for example, it is related to dopaminergic functions), it was hypothesized that caffeine could impact the specific symptoms of neurological disorders comprising this neurotransmitter [14]. It could also represent a target for the development of therapeutic plans addressing diverse neuropsychiatric conditions (including ADHD). Within this research area, the study of Vázquet et al. addressed a systematic review of scientific studies focused on the underlying effects of caffeine consumption on treating ADHD-like symptoms in animal studies [15]. The combined results of the 13 selected works suggested the concrete benefit of caffeine treatment, increasing attention levels and improving learning capacity, memory ability and olfactory discrimination without altering blood pressure and body weight. The authors concluded that the cumulate evidence of this review, supported at the neuronal level in animal models, strengthens the hypothesis that the cognitive effects of caffeine could be positive for intervention within ADHD, particularly during adolescence. However, they also observed that some of the reviewed studies provided inconsistent results, particularly concerning data referring to caffeine effects on locomotor activity and impulsivity. Exploring clues to explain the effects of caffeine within ADHD treatment plans is a growing area that warrants further research.

## 3. Diet Intervention on Anxiety and Depression 

Studies have identified a strong link between persistent stress and several adverse effects on the body’s immune, neuroendocrine, cardiovascular and central nervous systems [16]. Untreated chronic stress could also lead to other serious mental disorders such as anxiety and depression [17], recognized as the most common psychopathological states with high prevalence in contemporary societies. With an impact rate of around 332 million people for depression and 264 million for anxiety, depression and anxiety are the main reasons for disability worldwide [18]. Unfortunately, their rising prevalence, published globally during the last two years and characterized by COVID-19 [19], has increased awareness surrounding these disorders, recognizing them as major contributors to the global burden of disease.

Medical intervention (benzodiazepines and antidepressants) plus cognitive behavioral therapy is considered the gold standard intervention for depression and anxiety states. However, in some cases, these treatments are not effective, induce severe side effects (mostly medications) or are not recommended due to the patients’ characteristics (for example, very young or older age, or high risk of interaction with other medications). This scenario guided the search for new interventions for assisting in the prevention and management of anxiety-depressive illnesses, with less risk of side effects. Since the prerequisite to developing adequate evidence-based clinical guidelines is to assimilate dietary patterns and identify the biological mechanisms of action of critical nutrients [20], studies within the nutritional psychiatry research area have established similar pathophysiology systems related to the onset and development of depression and anxiety. Both mental illnesses increase oxidative stress, heighten inflammatory markers and over-activate stress and neuroplasticity pathways, with the result of altered neurotransmission and other brain structural changes [21,22]. The microbiome–gut–brain axis was identified as a key mediating pathway for the biological processes (including hypothalamic–pituitary–adrenal axis, immune function, modulation of BDNF and serotonin neurotransmission) [23]. Compelling evidence has encouraged research into the complementary use of nutrition supplements plus dietary plans with the aim to impact the pathways implied in psychiatric disorders. Studies have also reported common dietary factors in the underlying processes of anxiety and depression; particularly diets or supplements that are high in antioxidants and anti-inflammatories (such as Vitamin C, Vitamin A, Polyphenol and beta-carotene) have shown an inverse relationship with depression and anxiety severity levels [24,25].

Dietary changes and nutritional supplementation may also be beneficial in improving treatment response and quality of life among patients with depression and anxiety. The comprehensive scoping review conducted by Aucoin and colleagues [26], with a full-text review of 1541 articles selected (comprising animal research, human observational/experimental studies and meta-analyses of randomized trials), observed that decreased anxiety levels were related to healthy nutrition practices. Concretely, the least severe anxiety states reported dietary patterns characterized by caloric restriction, breakfast consumption, a broad spectrum of micronutrients–macronutrients supplementation (such as minerals, trace elements, vitamins, essential fatty acids), use of probiotics and intake of a range of fruits and vegetables. On the other extreme of the anxiety continuum, the most impairing states were associated with a high fat/high sugar/high cholesterol/high trans-fat diet, unbalanced tryptophan and protein and a high intake of carbohydrates. Regarding the prospective studies assessing treatment efficacy for dietary interventions, this scoping study concluded that the elimination of inflammatory foods, nutritional supplements and increases in the intakes of fruits and vegetables could contribute to better therapy responses, but only in combination with exercise and the other psychiatric and psychological interventions.

The meta-analysis of Berthelot and colleagues selecting randomized controlled trials concluded that fasting interventions, compared to control groups, achieved lower anxiety and depression levels, and it was also useful to decrease body mass index levels [27]. The benefit on weight was also interpreted as relevant since overweight and obesity are common among patients with depressive and anxiety states [28], and this comorbid condition is related to poor therapy response. [29]. The treatments meta-analyzed by Berthelot also showed to be safe in patients with other comorbid physical conditions, including type-2 diabetes. However, results did not allow recommending the fasting intervention type according to the baseline profiles (diagnostic subtype and severity).

This set of results are consistent with established evidence regarding healthy eating patterns and improvements in anxiety and depression states and the benefit of complementing the classic treatments with dietary interventions according to the specific needs of each patient. The low cost and the high effectiveness of these complementary plans may also confer additional benefits to physical aspects of health [30]. 

## 4. Diet Intervention on the Autism Spectrum 

It is known that some mental health conditions, such as those included within the autism spectrum, could severely impact the appetite and food choices [31]. Autism or autistic spectrum disorder (ASD) constitutes a complex wide spectrum of neurodevelopmental conditions typically identified during the first years of life, which affects brain functions and particularly areas of communication skills and social interaction. Typical symptoms are poor eye contact, refusing to be held or cuddled, impaired talking capacities, repeated–compulsive behaviors, restricted interests, lack of enthusiasm in playing with other children and lack of imaginative play. These impairments affect eating habits, encompassing some unhealthy concerns [32]: (a) eating an inadequate amount of food (some children within the ASD spectrum may show difficulties focusing on one concrete taste for an extended time or for eating a complete meal); (b) high sensitivity to the taste, smell, color or texture of foods, companied with avoiding specific foods or whole food groups (ASD individuals may express strong food dislikes and limited food selection); (c) potential medication interactions, which can lower appetite and/or even affect the absorption of certain elements (such as minerals and vitamins); and (d) the tendency of some ASD individuals to decrease their food intake as the children move from infancy to toddlerhood and childhood. Other frequent behavioral problems related to eating are gorging/hoarding food in the mouth, gagging/vomiting due to unliked food, not wanting to sit for meals or refusing social eating habits, sniffing/inspecting their own and others’ foods and taking food from others’ plates. 

A current empirical study aimed to explore the dietary patterns and nutrient intakes of ASD children compared to a healthy control group [33] observed that the autism group was characterized by higher consumption of cereals and pasta and a smaller intake of lean meat and eggs compared to reference dietary guidelines. The consumption of fruit, vegetables and fish was similar between the groups (lower than expected for a balanced diet), while the amounts of fatty meat and derivates, snacks, sweets and baked goods confectionery was higher than expected in an adequate diet in both ASD patients and controls. This research also observed that less than one-half of the ASD children tolerated solid foods and that compared to the control group, nutrient intakes were higher for energy, fat and saturated fat and lower for fiber, iron, iodine and some vitamins. These results are consistent with other studies identifying inadequate micronutrient intakes for some minerals among individuals within the autistic spectrum [34]. These individuals could benefit from adequate monitoring of nutritional presence and, if necessary, introducing supplements into their diet [35].

However, most interventions used to treat dysfunctional eating behaviors within the ASD spectrum (such as escape extinction, fading techniques and positive reinforcement) are aimed to increase the volume of food consumed, and few consider increasing the variety of foods and addressing protein–energy–micronutrient deficiencies [36,37]. Other dietary restrictions imposed by parents/caretakers as a therapeutic tool aimed to improve behavior and gastrointestinal symptoms (such as gluten-free or casein-free diets) further intensify the dietary vulnerability of individuals with ASD and in turn, could represent a big barrier to balanced eating [38,39]. Because nutritional status is the consequence of complex mechanisms and interactions, and since micronutrient deficiencies can seriously interact with development (such as scurvy due to low vitamin intake or reduced bone growth due to low levels of calcium), it is essential to explore the effectiveness of multi-component interventions aimed to provide more balanced eating within ASD [40].

## 5. Conclusions

Problems related to nutrition are often overlooked in patients with common mental health disorders (such as depression, anxiety, ADHD and ASD) towards interventions focused on medication complemented by behavior/psychotherapy treatments. Current research within the nutritional psychiatry area provides evidence regarding the role of nutrition and diet on these psychiatric conditions and offers a basis for developing new evidence-based intervention plans from a multidisciplinary perspective. Given the multifaceted and complex nature of mental and neurodevelopmental problems, the onset at early ages of the child (particularly for ADHD and ASD) and its persistent presentation across development stages (from early childhood to older age), the findings of these works could also contribute to the elaboration of guidelines/recommendations for improving the caring capacity of healthcare practitioners and family caregivers. In the end, improving the nutritional status of the patient will contribute to the individuals’ wellbeing and facilitate better progression of medical conditions. 

However, the design of effective dietary plans is based on the existence of reliable and valid assessment tools. Unfortunately, the current evidence does not propose nutritional assessment instruments specifically developed for individuals with different mental disorders (such as depression, anxiety, ADHD and ASD). Nutritional psychiatric research warrants additional attention and effort, combining varied methodologies and analyzing larger groups (clinical and population-based samples). Therefore, based on the existing research, dietary markers (foods variety, nutrients intake, sensory issues, preferences/restrictions and dietary intakes), biochemical indexes (vitamins, proteins, minerals, carbohydrates, lipids and other essential nutrients) and anthropometric evaluation (length-for-age, weight-for-age, weight-for-length, head circumference and other age-related-developmental indexes) should be key components of these measurement tools.

As a final thought, the study of how nutrition and mental health are linked is a growing research area, and the results obtained so far are highly promising. The ultimate objective is to facilitate new strategies for improving the quality of life and health of people with mental illness and to prevent the onset, aggravation and negative impacts of diseases.

## Data Availability

No original data has been analyzed for this manuscript.
